# TREM2 marks tumor-associated macrophages

**DOI:** 10.1038/s41392-020-00356-8

**Published:** 2020-10-09

**Authors:** Kyohei Nakamura, Mark J. Smyth

**Affiliations:** QIMR Berghofer Medical Research Institute, Herston, 4006 QLD Australia

**Keywords:** Tumour immunology, Tumour immunology

Immunosuppressive myeloid cells are recognized as a key barrier for cancer immunotherapy. Using an integrated technology for single-cell RNA-sequencing and intracellular protein measurements, A recent paper published in *Cell* provides evidence that TREM2 was identified as a marker for tumor-associated macrophages (TAMs) and monocytes.^1^

Immune checkpoint inhibitors (ICIs) targeting CTLA-4 or PD-1/PD-L1 have contributed to improve clinical outcomes in some types of malignancies. However, a substantial proportion of patients show limited clinical benefits following ICI therapy. While tumor-intrinsic factors, such as tumor mutational burden, have been recognized as a key determinant for therapeutic responsiveness to ICIs, the immunosuppressive tumor microenvironment (TME) also critically contributes to therapeutic resistance by limiting the tumor infiltration and activation of effector lymphocytes. In this context, targeting tumor-infiltrating myeloid cells such as TAMs and myeloid-derived suppressor cells might be a rational approach to harness antitumor immunity, given their potent immunosuppressive activities and abundance in the TME. However, due to the heterogeneity of immunosuppressive myeloid cells, it is often challenging to design myeloid-directed immunotherapy.^2^ A recent paper published in *Cell* provides evidence that TREM2 (Triggering-Receptor-Expressed on Myeloid cells 2)-expressing myeloid subsets are key regulators of antitumor immunity.^1^

TREM2 is a member of the Ig-superfamily that transmits activation signaling via an adaptor protein DAP12.^3^ The functional and pathological significance of the TREM2 receptor is best demonstrated in the context of Alzheimer’s disease (AD), as loss-of-function variants in *TREM2* are associated with an increased risk of AD.^3^ TREM2 expressed on microglia cells play a critical role for maintaining metabolic fitness during physiological stress by sensing multiple ligands such as lipids, lipoproteins, and oligomeric amyloid-β, which contribute to modulate AD-associated neuropathology.^3^ In addition to microglia, the expression of TREM2 is observed in tissue-resident macrophages, such as macrophages in the hair follicle stem cell niche, adipose tissues, and bone (osteoclast).^3^ However, it remained largely unknown whether TREM2 was important in myeloid immunosuppression in the TME.

Recent advances in single-cell RNA-sequencing (scRNA-seq) technology has allowed us to understand the immune landscape of various types of malignancies, and the substantial heterogeneity of TAMs and monocytes has been revealed.^2^ Indeed, it is often technically difficult to identify bona fide immunosuppressive myeloid subsets by transcriptional profiling alone, given that their immunosuppressive activities might be determined by multiple factors, such as metabolic status, signaling pathways, and enzymatic activities. To overcome this limitation, Katzenelenbogen et al. developed a new technology for the parallel recording of scRNA-seq and intracellular protein activity, called INs-seq (an integrated technology for scRNA-seq and intracellular protein measurements).^1^ Using an optimized fixative for RNA preservation and intracellular protein staining, Katzenelenbogen et al. demonstrated that INs-seq approach could successfully characterize heterogeneous T-cell subsets (segregated by protein expression levels of FOXP3, ID2, and TCF7) or differential signaling activity of myeloid subsets in response to LPS (segregated by protein expression levels of phosphorylated p38 MAPK), supporting that the INs-seq approach is a useful method to perform transcriptional profiling integrated with intracellular markers.

Taking advantage of this platform, Katzenelenbogen et al. next aimed to characterize tumor-infiltrating immunosuppressive myeloid subsets. It is appreciated that the conventional M1/M2 classification has limitations to capture the status of TAMs; however, arginase-1 (Arg1), an enzyme that metabolically dampens T-cell responses by deprivation of arginine, has been recognized as one of the key hallmarks of M2-skewed macrophages.^4^ Thus, Katzenelenbogen et al. performed in-depth profiling of Arg1^+^ tumor-infiltrating myeloid cells in MCA205 fibrosarcoma tissues. The myeloid compartment was separated into two Arg1^+^ subsets (TAMs and monocyte-like cells) and four Arg1^−^ subsets. Intriguingly, there was a correlation between Arg1 expression and *Trem2*, and thus, Katzenelenbogen et al. showed that Arg1^+^Trem2^+^ TAMs and Arg1^+^Trem2^+^ monocytic cells represented key regulatory myeloid subsets.^1^ To further understand the role of Trem2, Katzenelenbogen et al. challenged wild-type mice and *Trem2*^−/−^ mice with MCA205 tumor cells. Indeed, *Trem2*^−/−^ mice were significantly protected from tumor progression, which was associated with high infiltration of cytotoxic lymphocytes and NK cells and reduction of dysfunctional CD8 T cells.^1^ Together, the INs-seq approach successfully revealed a novel marker for tumor-infiltrating immunosuppressive myeloid cells, highlighting that the INS-seq is a powerful tool that confers an important layer of information to transcriptional profiling of heterogeneous population.

**Fig. 1:**
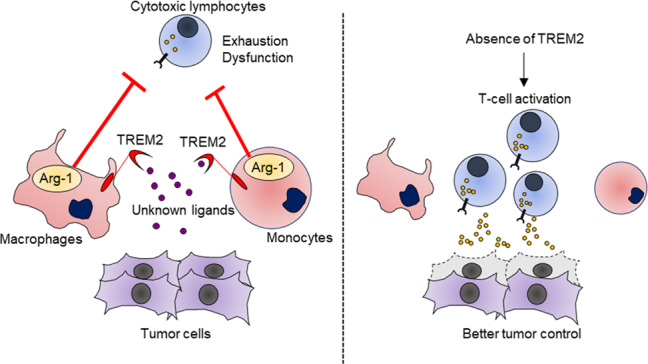
TREM2 is a key target to modulate tumor-associated macrophages (TAMs). TREM2 marks tumor-associated monocytes/macrophages with potent immunosuppressive activity by arginase-1 (Arg1) (left). The absence of TREM2 dramatically alters the immune microenvironment, leading to better antitumor immunity (right)

This work by Katzenelenbogen et al. elegantly demonstrated that TREM2 was a key phenotypic marker for TAMs and monocytes with potent immunosuppressive activity (Fig. 1). Concurrent work by Molgora et al. provides robust evidence that TREM2 is a potential therapeutic target to modulate immunosuppressive TAMs.^5^ TREM2 is known to recognize wide range of ligands, including lipoproteins, anionic lipids, and amyloid-β; however, it remains largely unknown what types of ligands that TREM2 recognizes in the TME. Notably, various macrophage scavenger receptors such as MARCO, SR-A, or Clever-1 are implicated in immunosuppressive activities of TAMs.^2^ Similarly, TAM receptors (i.e, Tyro3, Axl, and Mer) are also known to favor the generation of M2-like macrophages in the TME. It is possible that TREM2, together with these receptors, contributes to the functional maturation of TAMs by sensing various ligands released in the TME, such as lipids, lipoproteins, and apoptotic debris. Functional redundancy and non-redundancy among these receptors will require further investigation to determine the optimal therapeutic approach to redirect TAMs. Second, given that TREM2 can be released as a soluble form,^3^ the biological and clinical significance of soluble TREM2 in the context of cancer might also reveal additional roles of TREM2. Indeed, soluble CD163 (a scavenger receptor expressed on M2-like macrophages) has been studied to predict prognosis or therapeutic responsiveness to ICIs. Combining multiple soluble forms of TAM markers might allow us to understand the immunosuppressive status in cancer patients. Lastly, for clinical translation, it is critical to understand the therapeutic efficacy of TREM2 blockade in combination ICIs. Moreover, it is also worthwhile to investigate the therapeutic potential of TREM2 blockade in metastatic brain cancers or primary brain tumors, as these tumors might contain abundant TREM2-expressing microglia and monocyte-derived TAMs.

In summary, the INS-seq approach developed by Katzenelenbogen et al. defined bona fide immunosuppressive TAMs/monocytes among heterogeneous tumor-infiltrating myeloid cells, providing novel insight into the understanding of myeloid immunosuppression in cancers. Moreover, given that the strong impact of TREM2 on macrophage functional polarization, further studies are warranted to understand the role of TREM2 in chronic inflammatory diseases such as atherosclerosis, obesity, and carcinogenesis.
